# Evaluation of Ectopic Mitochondrial DNA in HeLa Cells

**DOI:** 10.3390/cimb44030080

**Published:** 2022-03-02

**Authors:** Mohammad T. Hussan, Noriko Matsui, Hideaki Matsui

**Affiliations:** 1 Department of Neuroscience of Disease, Brain Research Institute, Niigata University, Niigata 951-8585, Japan; tufazzal84@gmail.com (M.T.H.); noriyst2011@yahoo.co.jp (N.M.); 2 Department of Anatomy and Histology, Patuakhali Science and Technology University, Barishal 8210, Bangladesh

**Keywords:** ectopic DNA, mitochondrial DNA, mitochondrial transcription factor A (TFAM), Parkinson’s disease

## Abstract

The presence of ectopic DNA in the cytoplasm induces inflammation and cell death. It has been widely reported that leakage of nuclear DNA into the cytoplasm can mainly be sensed by cyclic GMP-AMP synthase (cGAS). We recently reported that mitochondria-derived cytoplasmic double-stranded DNA (dsDNA) that has escaped lysosomal degradation induces significant cytotoxicity in cultured cells and in vivo. Cytoplasmic mitochondrial DNA is assumed to be involved in various diseases and disorders, and more and more papers have been published confirming this. On the other hand, the current method for evaluating mitochondrial DNA in the cytoplasm may not be quantitative. Here, we introduce in detail a method to evaluate ectopic mitochondrial DNA in cells. This method is useful in basic research as well as in the study of aging, Parkinson’s disease, Alzheimer’s disease, heart failure, autoimmune diseases, cancer, and other conditions.

## 1. Introduction

DNA contains important information for survival and inheritance, and it needs to be protected from various stressors, including oxidants and mutagens. Eukaryotic cells contain mitochondria, which produce energy, together with a lot of active oxygen species, through aerobic respiration. Thus, in the eukaryotic cell, DNA exists in the nucleus or mitochondria, packed inside the nuclear membrane or mitochondrial membrane, respectively. However, DNA can be present in the cytoplasm under certain conditions, such as during viral or non-viral infections [1,2,3,4]. Even in some non-infectious conditions (for example, aging or various age-related disorders or cancer), DNA may be leaked into the cytoplasm from the nucleus or mitochondria [5,6,7]. DNA in the cytoplasm can be inflammatory and toxic to cells. It has been widely reported that leakage of nuclear DNA into the cytoplasm induces inflammation, cytotoxicity, and senescence-associated secretory phenotype (SASP), mainly via the cyclic GMP-AMP synthase (cGAS)-stimulator of the interferon genes (STING) pathway [5,6]. We recently reported that cytosolic double-stranded DNA (dsDNA) of mitochondrial origin escaping from lysosomal degradation was shown to induce cytotoxicity and neurodegeneration in cellular models of Parkinson’s disease [8]. The presence or increase in such ectopic DNA was often indicated by representative microscopic images and circumstantial evidence, not by the increase in DNA leakage itself. It is expected that the evaluation of this ectopic DNA will become more and more important, not only for basic research but also for research on aging and other diseases, such as Parkinson’s disease, Alzheimer’s disease, amyotrophic lateral sclerosis, heart failure, autoimmune diseases, diabetes, non-alcoholic steatohepatitis (NASH), and cancers. In fact, the cGAS–STING pathway has been reported to be crucial not only in aging but also in the pathogenesis of these diseases [8,9,10,11,12,13,14,15,16,17].

Probably due to the oxidative circumstances around the mitochondria and the lack of an efficient DNA-repair system for mitochondrial DNA, mitochondrial DNA is much more susceptible to mutation or damage than nuclear DNA [18,19]. Unlike nuclear DNA, mitochondrial DNA exists in multiple copies in a single cell and is synthesized as needed. Therefore, mitochondrial DNA might have a much greater chance of being leaked into the cytoplasm during aging or in malignant conditions such as age-related disorders, mitochondrial dysfunction, or cancers. Detection and measurement of such cytoplasmic mitochondrial DNA could be important in understanding the pathogenesis of various diseases and aging, and could possibly become a biomarker and a therapeutic target. We developed a semi-quantitative method to evaluate ectopic mitochondrial DNA leaking into the cytoplasm, and here we describe the method in detail. This paper enables objective evaluation of mitochondrial DNA leakage and is useful in the study of various disease pathologies.

## 2. Materials and Methods

### 2.1. Cell Lines, siRNA Treatment, and Quantitative PCR (qPCR) Analysis

HeLa cells were derived from cervical cancer cells from a female patient (ATCC, CCL-2). HeLa cells were cultured at 37 °C under 5% CO_2_ in Dulbecco’s Modified Eagle Medium (DMEM) (Nacalai Tesque, Kyoto, Japan) supplemented with 10% fetal bovine serum (FBS) (Biowest, Nuaillé, France) and a penicillin–streptomycin solution (100 units/mL penicillin G and 100 μg/mL streptomycin sulfate, Wako, Osaka, Japan). One day before transfection, HeLa cells were plated at a density of 600 cells/μL. The expression of genes of interest was silenced in HeLa cells using Stealth RNAi siRNA (mitochondrial transcription factor A, TFAM: HSS144251, glucocerebrosidase, GBA: HSS178140, Thermo Fisher Scientific, Waltham, MA, USA) in Lipofectamine RNAiMAX Transfection Reagent (Thermo Fisher Scientific) according to the manufacturer’s instructions (a final RNA concentration of 10 nM). Three days after siRNA knockdown, total cellular RNA was isolated using TRIzol reagent according to the manufacturer’s instructions (Thermo Fisher Scientific). The cDNA templates were synthesized from the purified RNA using the ProtoScript II First Strand cDNA Synthesis Kit (New England Biolabs, Ipswich, MA, USA) with oligo (dT)20 primers. qPCR was performed using TB Green Premix Ex Taq II (Takara Bio, Kusatsu, Japan) and analyzed in a Thermal Cycler Dice Real Time System Lite (Takara Bio). The PCR primers used in the present study were *GBA* forward (TGCTGCTCTCAACATCCTTGCC), *GBA* reverse (TAGGTGCGGATGGAGAAGTCAC), *TFAM* forward (GGCAAGTTGTCCAAAGAAACC), *TFAM* reverse (GCATCTGGGTTCTGAGCTTTA), *Glyceraldehyde-3-Phosphate Dehydrogenase (GAPDH)* forward (CAGCCTCAAGATCATCAGCA), and *GAPDH* reverse (TGTGGTCATGAGTCCTTCCA). Statistical analyses were performed using GraphPad Prism software version 9.3.1 (GraphPad Software, San Diego, CA, USA). Two-sided Student’s *t*-tests were used to compare arithmetic means between two groups. Data are presented as the means and ± standard errors of the means.

### 2.2. Immunofluorescence Detection of Ectopic Mitochondrial DNA and Image Analysis

One day before transfection, HeLa cells were plated at a density of 600 cells/μL. The expression of genes of interest was silenced in HeLa cells using Stealth RNAi siRNA (Thermo Fisher Scientific) in Lipofectamine RNAiMAX Transfection Reagent (Thermo Fisher Scientific) according to the manufacturer’s instructions (a final RNA concentration of 10 nM). Three days after siRNA knockdown, cells were subjected to immunofluorescence staining. For the detection of mitochondrial DNA, anti-dsDNA antibody (Abcam, Cambridge, UK, clone 35I9, Cat# ab27156, RRID: AB_470907 or EMD Millipore, Billerica, MA, USA, clone AC-30-10, Cat# CBL186, RRID: AB_11213573) showed a better signal-to-noise ratio than Hoechst 33258, DAPI, or Picogreen. Here we combined three primary antibodies: anti-dsDNA antibody (35I9 DNA) (Abcam, Cat# ab27156, RRID: AB_470907), anti-histone-H2B antibody (Abcam, Cat# ab134211), and anti-heat-shock-protein-60 (Hsp60) antibody (Abcam, Cat# ab46798, RRID: AB_881444). Other antibodies can be chosen or fluorescently tagged with histone, or Hsp60 can be used alternatively, but it is necessary to optimize the conditions of triple staining for each combination. Cells were washed twice with phosphate-buffered saline (PBS) and fixed with ice-cold methanol for 10 min at 4 °C. Alternatively, 4% paraformaldehyde (PFA) can be used for fixation, but the background signals seem much greater in PFA-fixed samples. After fixation, the cells were washed three times with phosphate-buffered saline with Tween 20 (PBST) for 5 min each. Cells were permeabilized with PBS containing 0.2% (*w/v*) Triton X-100 for 10 min. After being washed three times with PBST for 5 min each time, cells were incubated with 2% (*w/v*) bovine serum albumin (BSA) in PBST for 30 min. The cells were incubated with a single primary antibody, anti-histone-H2B antibody (1/400, Abcam, Cat# ab134211) diluted in 2% (*w/v*) BSA in PBST O/N at 4 °C. After being washed three times with PBST for 5 min each time, cells were incubated with an Alexa Fluor 488-AffiniPure Donkey anti-Chicken IgY (IgG) (H+L) (1/200, Wako, Cat# 563-78311) diluted in 2% (*w/v*) BSA in PBST for 1 h at room temperature (RT). After being washed three times for 5 min each time with PBST, cells were incubated with primary antibodies, and anti-dsDNA antibody (35I9 DNA) (1/800, Abcam, Cat# ab27156, RRID: AB_470907) and anti-Hsp60 antibody (1/400, Abcam, Cat# ab46798, RRID: AB_881444) diluted in 2% (*w/v*) BSA in PBST were applied for 1 hr at RT or overnight (O/N) at 4°C. After being washed three times with PBST for 5 min each time, cells were incubated with highly cross-adsorbed Alexa Fluor 680-conjugated Donkey anti-Rabbit IgG (H+L) (1/200, Thermo Fisher Scientific, Cat# A10043, RRID: AB_2534018) and Donkey anti-Mouse IgG H&L (Alexa Fluor^®^ 594) (1/200, Abcam Cat# ab150108, RRID: AB_2732073) diluted in 2% (*w/v*) BSA in PBST for 1 hr at RT. The cells were washed three times with PBST for 5 min each time, and the specimen was analyzed using an A1R+ confocal microscope (Nikon, Tokyo, Japan).

The microscopic images were then subjected to image calculation using ImageJ software (National Institutes of Health, Bethesda, MD, USA). The signal of Hsp60 was multiplied by “x”. The “x” value was determined as follows: subtracting the “x”-folded Hsp60 signal from the mitochondrial DNA signal in the control group made the mitochondrial-DNA value less than or equal to zero. The signal of histone H2B was multiplied by “y”. The “y” value was determined as follows: subtracting the “y”-folded histone-H2B signal from the nuclear-DNA signal in the control group made the nuclear-DNA value less than or equal to zero. If the background signal becomes high from the multiplication, the contrast of the multiplied image can be adjusted for the same conditions in all the samples. The image of dsDNA staining, minus the adjusted image of multiplied Hsp60 and histone H2B, was used for subsequent analysis. Using the “Adjust-Threshold” function, these images were binarized. We selected dsDNA puncta ranging in size from 2 to 20 μm^2^ (circularity 0.1–1.0), and they were counted using the “Analyze-Analyze Particles” function.

### 2.3. Electron Microscopy

One day before transfection, HeLa cells were plated at a density of 600 cells/μL. The expression of genes of interest was silenced in HeLa cells using Stealth RNAi siRNA (Thermo Fisher Scientific) in Lipofectamine RNAiMAX Transfection Reagent (Thermo Fisher Scientific) according to the manufacturer’s instructions (with a final RNA concentration of 10 nM). Four days after siRNA knockdown, cells were subjected to fixation. Cell-culture samples were fixed with 2% PFA and 2% glutaraldehyde in 0.1 M phosphate buffer, pH 7.4, at 37 °C, then placed in a 4 °C refrigerator for 30 min. The samples were fixed in 2% glutaraldehyde in 0.1 M phosphate buffer overnight at 4 °C. The samples were washed three times with 0.1 M phosphate buffer for 30 min and post-fixed with 2% osmium tetroxide in 0.1 M phosphate buffer at 4 °C for 1 hr. The samples were dehydrated in graded ethanol solutions, transferred to resin (Quetol-812, Nisshin EM Co., Tokyo, Japan), and polymerized at 60 °C for 48 h. The polymerized resins were cut into ultrathin 70-nm sections with a diamond knife using an ultramicrotome (Ultracut UCT, Leica Microsystems, Wetzlar, Germany) and were then mounted on copper grids. The sections were stained with 2% uranyl acetate at RT for 15 min and washed with distilled water, followed by secondary staining with a lead staining solution (Sigma-Aldrich, Tokyo, Japan) at RT for 3 min. The grids were observed under a transmission electron microscope (JEM-1400Plus, JEOL Ltd., Tokyo, Japan) at an acceleration voltage of 80 kV (zebrafish brain) or 100 kV (cell), and images were recorded using a charge-coupled-device (CCD) camera (EM-14830RUBY2, JEOL Ltd.).

### 2.4. Immunoelectron Microscopy

The samples on the gold disks were frozen in liquid propane at −175 °C. After the samples were frozen, they were freeze-substituted with 1% tannic acid in ethanol and 2% distilled water at −80 °C for 24 hr. The samples were then kept at −20 °C for 4 hr, followed by incubation at 4 °C for 1 hr. Next, the samples were dehydrated in anhydrous ethanol 3 times for 30 min. each time, followed by infiltration with a 50:50 mixture of ethanol and resin (LR white: London Resin Co. Ltd., Berkshire, UK) at 4 °C for 1 hr. The samples were transferred to fresh 100% resin and incubated at 4 °C for 30 min, and this process was repeated three times. The samples were transferred to fresh 100% resin and were polymerized at 50 °C O/N. The polymerized resins were ultra-thin sectioned at 90 nm with a diamond knife using an ultramicrotome (Ultracut UCT; Leica), and the sections were placed on nickel grids. The grids were incubated with anti-dsDNA antibody (35I9 DNA) (1/800, Abcam, Cat# ab27156, RRID: AB_470907) in 1% BSA/PBS at 4 °C O/N, followed by three rinses with 1% BSA/PBS for 1 min each. They were subsequently incubated with the secondary antibody conjugated to 15-nm gold particles (Goat anti-Mouse IgG polyclonal antibody) for 2 hr at RT. After rinsing with PBS, the grids were placed in 2% glutaraldehyde in 0.1 M cacodylate buffer. Afterward, the grids were dried and then were stained with 2% uranyl acetate for 15 min and with a lead stain solution (Sigma-Aldrich) at RT for 3 min. The grids were observed through a transmission electron microscope (JEM-1400plus; JEOL Ltd.) at an acceleration voltage of 100 kV. Digital images were obtained with a CCD camera (EM-14830RUBY2, JEOL Ltd.).

## 3. Results

### 3.1. Decreased TFAM or GBA Induces an Increase in Ectopic Mitochondrial DNA

**Figure 1:**
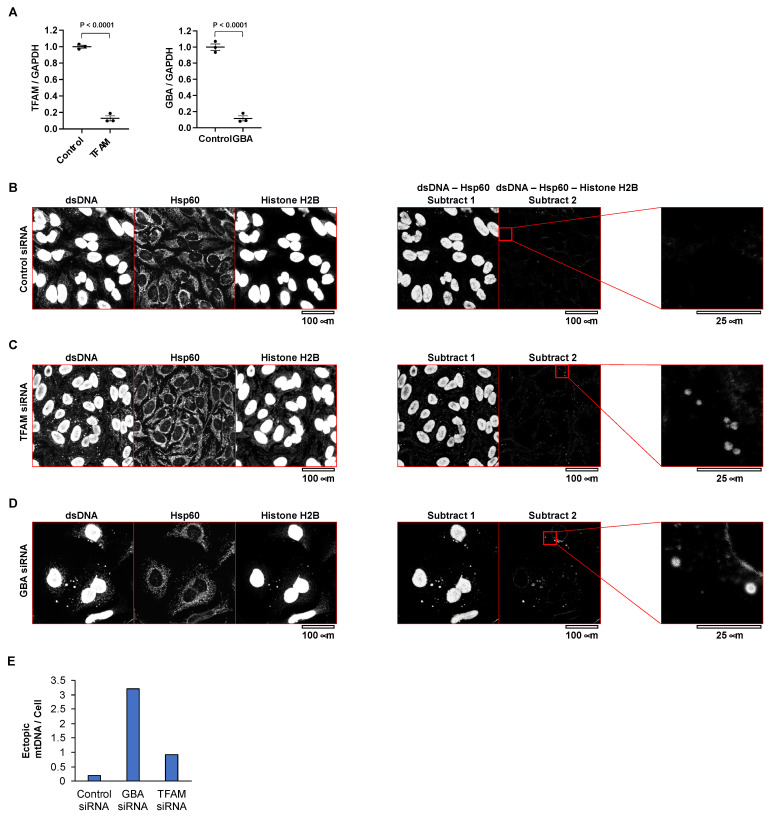
Loss of TFAM or GBA protein leads to cytosolic leakage of mitochondrial DNA. (**A**) qPCR of *TFAM* and *GBA* mRNA 3 days after siRNA transfection. (**B**) Immunostaining for dsDNA, histone H2B, and Hsp60 in HeLa cells transfected with control siRNA (day 3). Subtract-1 image shows the subtraction of the Hsp60 image from the dsDNA image. Subtract-2 image shows the subtraction of the Hsp60 image and histone-H2B image from the dsDNA image. The image on the far right is an enlargement of the red square in the Subtract-2 image. (**C**) Knockdown of TFAM expression with siRNAs in HeLa cells. Immunostaining for dsDNA, histone H2B, and Hsp60 in HeLa cells transfected with TFAM siRNA (day 3). Subtract-1 image shows the subtraction of the Hsp60 image from the dsDNA image. Subtract-2 image shows the subtraction of the Hsp60 image and istone-H2B image from the dsDNA image. The image on the far right is an enlargement of the red square in the Subtract-2 image. (**D**) Knockdown of GBA expression with siRNAs in HeLa cells. Immunostaining for dsDNA, histone H2B, and Hsp60 in HeLa cells transfected with GBA siRNA (day 3). Subtract-1 image shows the subtraction of the Hsp60 image from the dsDNA image. Subtract-2 image shows the subtraction of the Hsp60 image and histone-H2B image from the dsDNA image. The image on the far right is an enlargement of the red square in the Subtract-2 image. (**E**) The bar graph shows the ratio of ectopic mitochondrial DNA dots per cell number among HeLa cells transfected with control, TFAM, and GBA siRNAs.

**Figure 2:**
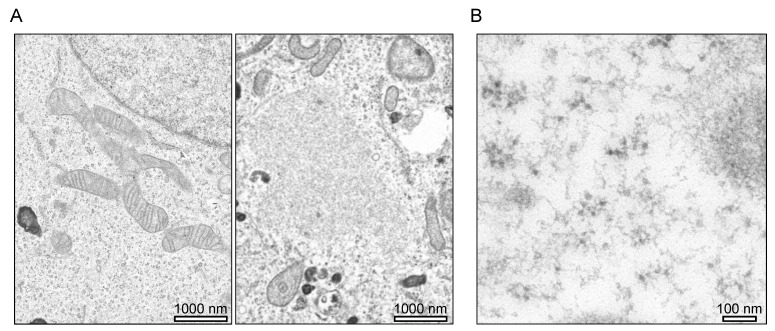
Electron micrographs of HeLa cells transfected with TFAM siRNA (day 4). (**A**) The image on the right shows HeLa cells with TFAM siRNA, and the image on the left shows HeLa cells without TFAM siRNA. TFAM siRNA-transfected HeLa cells show aggregates with no membrane structure and disrupted mitochondria. (**B**) Detection of dsDNA by anti-dsDNA antibody (35I9 DNA) and 15-nm gold particles. Signals of dsDNA are shown as dark spots.

First, we showed an example of the detection of mitochondria-derived cytoplasmic DNA in HeLa cells in which the TFAM protein was knocked down by siRNA (Figure 1A). TFAM is a key mitochondrial transcription factor and also functions in mitochondrial DNA replication and repair [20]. Sequence polymorphisms in this gene are reported to be associated with Parkinson’s disease [21,22]. After 3 days of TFAM protein knockdown, a lot of double-stranded DNA dots were detected in the cytoplasm of the HeLa cells. Histones are positive markers for nuclear-origin DNA in the cytoplasm, and negative markers for mitochondrial DNA and mitochondrial-origin DNA in the cytoplasm [23]. These dots did not co-localize with histones, suggesting that they were not nuclear-derived DNA. Furthermore, some of these cytoplasmic, ectopic DNA dots did not co-localize with Hsp60, a marker for the mitochondrial matrix, indicating that they resided outside the mitochondria (Figure 1B,C). Electron microscopy also showed that the ectopic DNA leaked from the mitochondria (Figure 2A,B). In summary, the increase in ectopic DNA dots in the cytoplasm by TFAM knockdown was due to mitochondrial DNA leaking from the mitochondria.

Second, we showed another example of the detection of mitochondria-derived cytoplasmic DNA in HeLa cells in which GBA protein was knocked down by siRNA (Figure 1A). *GBA* is the causative gene for Gaucher disease, but heterozygous mutation of *GBA* is also a risk factor for Parkinson’s disease [24]. A lot of double-stranded DNA dots were detected in the cytoplasm of the HeLa cells after 3 days of GBA protein knockdown. Some DNA dots were co-localized with histones, suggesting that they were nuclear-derived DNA. Other DNA dots were not co-localized with histones, suggesting that they were not nuclear-derived DNA but mitochondria-derived DNA (Figure 1D). In summary, the increase in ectopic DNA dots in the cytoplasm by GBA knockdown was at least partly due to mitochondrial DNA leaking from the mitochondria. 

### 3.2. Semi-Quantitative Evaluation of Ectopic Mitochondrial DNA

Images of Hsp60 staining (the mitochondrial matrix) and histone-H2B staining (the nuclear marker) were subtracted from images of dsDNA staining. Then, using the “Adjust-Threshold” function of the ImageJ software (National Institutes of Health), images were binarized. We selected dsDNA puncta ranging in size from 2 to 20 μm^2^ (circularity 0.1–1.0) using the “Analyze-Analyze Particles” function. If necessary, the original images were examined for the final count. If the signal of histone H2B was small, then the subtracted images showed donut-like structures. These represent cytosolic dsDNA of nuclear origin and should be eliminated from the quantity of cytosolic dsDNA of mitochondrial origin. The range of size can be modified according to the cell type or the origin of histological specimens. Figure 1E shows an example of the counting of ectopic mitochondrial DNA dots using 1B–D.

Alternatively, images of Hsp60 staining were subtracted from images of dsDNA staining. Then, the number of dsDNA puncta was counted using the “Adjust-Threshold” and “Analyze-Analyze Particles” functions. Subsequently, dsDNA puncta without the histone-H2B signal were counted by direct observation. The latter method is suitable when the histone-H2B images show a low signal-to-noise ratio. 

Altogether, this method can clearly show ectopic mitochondrial DNA leaking into the cytoplasm and allows an objective and statistical evaluation of such ectopic mitochondrial DNA in cells.

## 4. Discussion

In our study, we developed a method that enables the counting of cytoplasmic mitochondrial DNA. Detection of cytoplasmic mitochondrial DNA has been reported by using immunofluorescence techniques, but the methods were not quantitative for the evaluation of cytoplasmic mitochondrial DNA in most cases [15,25]. In another study, droplet PCR was conducted to quantitate circulating mitochondrial DNA [9]. Recently, Sato et al. [26,27] published an excellent protocol to evaluate cytoplasmic DNA by immunofluorescence microscopy using Lamin B1 (LMNB1) antibodies as a nuclear structure marker and cytochrome c oxidase subunit 4 (COX4) as a mitochondrial structure marker. This protocol seems very useful for quantitating cytoplasmic dsDNA of nuclear origin. However, when we tried COX4 or other mitochondrial membrane markers, including both inner and outer markers, it was very difficult to precisely discriminate the cytoplasm from the mitochondria. For example, if we observed circular signals using a mitochondrial membrane marker and observed dsDNA signals within, then this dsDNA could represent either mitochondrial DNA inside the circular mitochondria (normal) or cytoplasmic mitochondrial DNA outside the donut-like mitochondria (ectopic). We thus used Hsp60 as a marker labeling the mitochondrial matrix to evaluate the ectopic mitochondrial DNA in the cytoplasm.

As described in a recent review [23], there are many different types of cytoplasmic dsDNA of nuclear origin, and most of them are positive for histone markers. Due to the limitation of the combination of antibodies used for triple staining, we used histone H2B as a histone marker. However, if this limitation is solved, γH2AX may be a better marker for cytoplasmic dsDNA of nuclear origin. Histone-H2B-negative cytoplasmic dsDNA was seldom observed in the control HeLa cells used in this study, but such dsDNA of nuclear origin may be present if other cell lines or conditions are used.

The in situ hybridization of mitochondrial DNA used in our recent paper is suitable for qualitative evaluation, but it might be difficult to utilize in situ hybridization for quantitative evaluation [8]. In situ hybridization exposes the specimen to high temperature, which can result in deformation or noise of some parts of the specimen. On the other hand, the immunocytochemical method presented in this paper can obtain signals with low background noise.

In our recent paper, in the human cells and zebrafish used as Parkinson’s disease models, we showed that mitochondrial DNA was leaked out into the cytoplasm, which is toxic to the cells. We also observed that the quantity of cytosolic mitochondrial DNA was increased in the post mortem brain tissues of patients with Parkinson’s disease [8]. These lines of evidence suggest that an increased quantity of ectopic mitochondrial DNA leaked out from the mitochondria can induce acute and chronic inflammation resulting in various diseases, including Parkinson’s disease and heart failure; thus, upregulation of cytosolic-DNA degradation or inhibition of its sensors could be a potential therapeutic target for these diseases [8,28]. The method introduced in this paper for the quantitative evaluation of ectopic mitochondrial DNA serves as an important tool for further research in this field.

## Data Availability

The data and tools described in this manuscript are available upon request.
